# Reverse shoulder arthroplasty used for revision of reverse shoulder arthroplasty: a systematic review

**DOI:** 10.1016/j.xrrt.2021.07.002

**Published:** 2021-08-18

**Authors:** John J. Heifner, Anjali D. Kumar, Eric R. Wagner

**Affiliations:** a St George’s School of Medicine, Great River, NY, USA; b Gillings School of Global Public Health, University of North Carolina at Chapel Hill, Chapel Hill, NC, USA; c Division of Upper Extremity Surgery, Department of Orthopaedic Surgery, Director of Upper Extremity Surgery Research, Atlanta, GA, USA

**Keywords:** Failed reverse shoulder, Glenoid bone loss, Reverse shoulder replacement, Revision reverse shoulder, Revision shoulder arthroplasty

## Abstract

**Background:**

As reverse shoulder arthroplasty (RSA) cases increase, so too will the need to revise subsequent failures. Many of the complications associated with revising anatomic total shoulder and hemiarthroplasty have been adequately addressed by RSA including glenoid bone deficiency, instability, and functional outcomes. However, the risk for complication when revising a failed reverse prosthesis may be more pronounced with increased bone and soft tissue deficiency. The ability for the reversed prosthesis to accommodate these insufficiencies following a prior reversed prosthesis is unclear.

**Methods:**

PubMed, Embase, and Google Scholar were queried for articles which fit the inclusion criteria of a reversed prosthesis used to revise a failed primary reverse prosthesis with a minimum follow-up of 12 months and clinical outcome reporting.

**Results:**

After exclusions, 9 studies reporting on 242 reverse shoulders with a mean follow-up of 40.29 months were analyzed. The differences between preoperative and postoperative weighted means were not significant for Constant (*P* = .26), American Shoulder and Elbow Surgeons Shoulder score (*P* = .61), SSV (*P* = .57), and visual analog scale for pain (*P* = .48). Functional improvements in elevation (74°-102°) and external rotation (18°-21°) were consistent with those reported for primary reverse procedures, although differences in preoperative and postoperative measures were not statistically significant. Patient satisfaction was 89% with a major complication rate of 25%.

**Discussion:**

The reverse shoulder prosthesis has proven satisfactory in revising hemiarthroplasty and anatomic total shoulder arthroplasty. The current results indicate RSA is also a satisfactory treatment option when revising a prior reverse prosthesis. Inherent to revision shoulder surgery is the obstacle of humeral and glenoid bone loss, an attenuated soft-tissue envelope, and instability. The reverse prosthesis may adequately address these commonly confronted difficulties with its inherent design characteristics. RSA provides a secure glenoid fixation for bone grafting, the ability to increase construct stability with component sizing, and a reliance on the deltoid for function. As our learning about revision of RSA improves, so will our ability to preemptively address potential issues which may lead to decreased complications in these cases. Despite the 25% rate of major complication, patients reported satisfaction of 89% which demonstrates the improvements in function and pain relief that are provided by the reverse prosthesis.

With the precipitous rise of reverse shoulder arthroplasty (RSA),^16^^,^^17^^,^^29^^,^^54^ comes the eventual need to address RSA failure with revision surgery. Reported outcomes for reverse shoulder replacement have been favorable for the primary conditions of cuff tear arthropathy,^20^^,^^42^^,^^51^^,^^52^ osteoarthritis,^37^^,^^43^^,^^51^ fracture sequelae,^7^^,^^8^^,^^10^^,^^11^^,^^25^ and for revision of hemiarthroplasty and anatomic shoulder replacement.^3^^,^^46^^,^^48^^,^^51^ According to a recent analysis, revision shoulder arthroplasty has increased concurrently with primary arthroplasty over the past 20 years.^21^ With the projected rise in primary shoulder arthroplasty,^48^ the expectation is that revision arthroplasty will experience a consequential rise.

A recent multicenter review of revision shoulder arthroplasty showed that RSA was used as the revision implant in 48% of all revision cases and in 75% of all RSA revision cases.^21^ The authors state the reliability of RSA when revising hemiarthroplasty and anatomic total shoulder arthroplasty is due to its ability to address bony and soft-tissue deficiency. Whether these capacities lead to favorable performance when RSA is used to revise an RSA is unknown.

Potential salvage options after a failed reverse prosthesis include resection arthroplasty or hemiarthroplasty which have been historically associated with poor outcomes due in part to the lack of soft-tissue and bony constraints.^3^^,^^13^^,^^28^^,^^30^^,^^39^^,^^49^ With the improved technology and surgical techniques of RSA, revision of hemiarthroplasty and anatomic shoulder replacement have improved short-term outcomes in recent years.^7^^,^^19^^,^^35^^,^^50^ However, even with these improved outcomes, revision shoulder arthroplasty is still associated with a high rate of complications. The risk for complications can be especially problematic when revising a failed RSA as these cases can have even more pronounced bony and soft-tissue deficiencies, often requiring grafting or other salvage procedures.^5^^,^^9^^,^^45^^,^^47^^,^^49^^,^^50^^,^^53^

In recent years, studies have examined the outcomes of RSA being revised to RSA. Therefore, the purpose of this systematic review was to analyze pooled clinical outcomes of RSA being used to revise a failed RSA. Furthermore, we sought to determine whether the preoperative indication for revision held prognostic value for further complication. We hypothesize that these procedures can yield reasonable functional outcomes albeit with high rates of complication.

## Materials and methods

A broad search of the literature was performed for all peer-reviewed studies published before July 2019. The databases PubMed, Embase, and Google Scholar were queried using the keywords “revision OR revised reverse shoulder arthroplasty OR prosthesis” and “failure of reverse shoulder arthroplasty OR prosthesis”.

### Study selection

**Figure 1:**
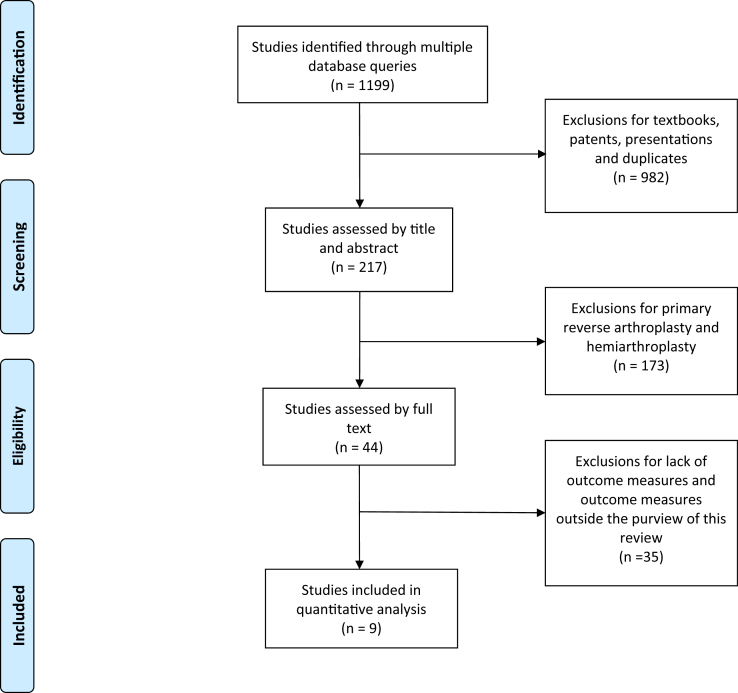
Flowchart depicting literature search protocol with exclusion criteria and final study selection.

The search returned a total of 1199 studies with 217 being screened via abstract and title. In addition, references of included studies and recent reviews were screened for potentially relevant studies. Inclusion required (1) full-text peer-reviewed publication, (2) patients with a failed primary reverse prosthesis which was revised to a reverse prosthesis, (3) reporting of clinical outcomes, and (4) a minimum of 12-month follow-up. Exclusion criteria included (1) primary reverse shoulder arthroplasty, (2) revision surgery using means other than RSA, and (3) outcome measures outside our purview. Figure 1 outlines the database search result and study selection process.

### Data collection

Research characteristics included type of study, year of publication, number of patients, and follow-up duration. Patient demographics included age and gender. Surgical data included indication for revision, glenoid bone grafting, humeral stem cementation, repair of the subscapularis, and prosthesis manufacturer. Outcome measures included patient-reported scores and satisfaction, range of motion, rates of complication, and subsequent revision of the prosthesis.

### Methodological quality

We assessed quality of evidence with the 100-point Modified Coleman Methodology Score (MCMS) which contains 17 variables across 2 sections. The Coleman score addresses the inherent study design as well as the quality of outcome reporting thus producing a comprehensive measure for critiquing research. Section A analyzes the elemental structure of the study via sample size, follow-up interval and treatment description. Section B analyzes the strength of the conclusion via the presence of patient-reported outcomes and the retention of subjects.

### Outcome measures

Patient-reported outcome measures included Constant, American Shoulder and Elbow Surgeons Shoulder score, visual analog scale for pain, Simple Shoulder Test, subjective shoulder value, and patient satisfaction. Forward elevation, abduction, and external rotation comprised the functional data. Major complications, minor complications, and rates of revision were recorded. We classified major complications as any component-related mechanism, instability, periprosthetic fracture, and deep infection. Minor complications included all nonmajor and medical complications.

### Statistical analysis

Statistical analysis was conducted using demographic information, pain outcomes, functional scores, device implant information, and complication data from 9 studies. Pooled means of the data (age, follow-up, visual analog scale, American Shoulder and Elbow Surgeons Shoulder, Constant, subjective shoulder value, elevation, abduction, and external rotation) were used and frequency-weighted to represent the number of patients that participated in each study. All other data (gender, device information, and complications) were summed as pooled frequency counts. The differences in preoperative and postoperative frequency-weighted outcome means were compared using two-sample, two-tailed t-tests with unequal variances with an alpha significance level of *P* < .05.

## Results

The 9 included studies reported a total of 242 revision reverse implants with a mean patient age of 69 (range 62-73) years and a mean follow-up of 40.3 months (range 24-59). (Table I) Indications for revision surgery are shown in Table II. Patient-reported outcomes and functional parameters are detailed in Table III with no significant differences between preoperative and postoperative values. Pooled mean for patient satisfaction was 89% across the 5 studies (55%) that reported it. The use of glenoid bone graft was reported for 32 patients (13%) across 5 studies. Indications for the primary reverse procedure and rates of additional revision after the RSA revision are detailed in Table IV. MCMS across all 9 studies was 59.9 which indicate a moderate methodological quality.

**Table I:** Patient and research characteristics for all included studies.

Average age at surgery, yr (range)	69 (62-73)
Percent female, n (%)	46 (48%)
Average follow-up, mo (range)	40.29 (24-59)
Total reverse shoulder arthroplasty	242

**Table II:** Indications for revision of reverse shoulder arthroplasty.

Indication	n, (%)
Glenoid component failure	60, (38%)
Infection	42, (26%)
Instability	23, (14%)
Dislocation	18, (11%)
Humeral component failure	15, (9%)

**Table III:** Preoperative and postoperative values for clinical results.

Outcome	Shoulders, n (%)	Preoperative∗	Postoperative∗	*P* value
Functional Outcomes				
Elevation, °	159 (66%)	74.1	102.3	.59
Abduction, °	99 (41%)	60.0	101.1	.57
External Rotation, °	158 (65%)	17.8	20.7	.84
Patient-reported outcomes				
VAS for pain	102 (42%)	5.55	2.92	.48
ASES	143 (59%)	41.37	59.96	.60
Constant	127 (52%)	25.97	48.38	.26
SSV	76 (31%)	36.72	54.85	.67

*ASES*, American Shoulder and Elbow Surgeons score; *SSV*, subjective shoulder value; *VAS*, visual analog scale.

Statistical significance at *P* < .05.

∗ Data presented as frequency weighted mean.

**Table IV:** Indications for primary reverse shoulder arthroplasty and rates of additional revision after revision of the primary reverse prosthesis.

Study	Indication for primary reverse shoulder arthroplasty						Revision RSA outcome	
	CTA	MIRCT	FRCR	Trauma sequelae	Revision of HA/TSA	Other	Additional revision	Patient satisfaction
Black^3^	8 (50%)	3 (19%)			5 (31%)		44%	89%
Holcomb^26^	9 (64%)		4 (29%)		1 (7%)		14%	100%
Boileau^5^	5 (16%)		4 (13%)	7 (22%)	12 (38%)	4 (13%)	#36∗	89%
Beekman^2^	8 (73%)					3 (27%)	9%	NR
Ladermann^30^	12 (44%)	8 (30%)		5 (19%)	2 (7%)		NR	83%

*CTA*, cuff tear arthropathy; *MIRCT*, massive irreparable rotator cuff tear; *FRCR*, failed rotator cuff repair; *HA/TSA*, hemiarthroplasty, total shoulder arthroplasty; *NR*, not reported or not able to extrapolate data for study population; *RSA*, reverse shoulder arthroplasty.

∗ 36 additional revision procedures across 32 patients.

### Complications

Of the 9 studies analyzed, 5 studies reported complication details at a mean follow-up of 41 months. (Table V) These 5 studies represented 39% (n = 95) of the total number of cases (n = 242) analyzed in this review. Major complications reported across these studies included dislocation (7), glenoid loosening (5), periprosthetic fracture (4), instability (3), deep infection (2), polyethylene fracture (1), and component dissociation (1) equating to a major complication rate of 24.6%. Subsequent revision rate of 22.1% (N = 15) was reported by 4 studies. Minor complications were reported in 3 studies (33%) – hematoma (2) and superficial infection (1).

**Table V:** Research characteristics and clinical outcomes for all include studies.

Study	RSAs	∗CS	∗ASES	∗Elevation, °	∗ER, °	Major complications	% Major Complications	Patient satisfaction
Black^3^	16	NR	66.7	NR	NR	3 instability, 3 GL, 2 Pp Fx	50%	89%
Middernacht^36^	29	60	NR	NR	NR	NR	NR	NR
Holcomb^26^	14	NR	70	118	22	1 GL, 1 dislocation	14%	100%
Boileau^5^	32	47	NR	111	7	NR	NR	89%
Wagner^49^	27	NR	66	NR	38	5 dislocation, 2 Pp Fx, 1 DI, 1 GL, 1 poly fx, 1 dissociation	41%	86%
Wiater^53^	28	38	59.9	90	NR	NR	NR	NR
Stephens^44^	58	NR	52.9	97	25	NR	NR	NR
Beekman^2^	11	55	NR	NR	NR	1 dislocation, 1 DI	18%	NR
Ladermann^30^	27	45.6	NR	108	10	NR	0%	83%

*ASES*, American Shoulder and Elbow Surgeon score; *CS*, Constant score; *DI*, deep infection; *ER*, external rotation; *GL*, glenoid loosening; *NR*, not reported or not able to extrapolate data for the study population; *RSA*, reverse shoulder arthroplasty; *Pp Fx*, periprosthetic fracture; *poly fx*, polyethylene fracture.

∗ Data presented as frequency weighted mean.

## Discussion

Adequate data exist for outcomes of RSA used in revision of failed anatomic prostheses and for failed hemiarthroplasty.^3^^,^^7^^,^^19^^,^^35^^,^^46^^,^^48^^,^^50^^,^^51^ However, with the increased volume of primary RSA,^17^^,^^29^ evidence is needed to determine acceptable treatments for failed reverse implants.

We calculated a 25% major complication rate across the 5 studies which detailed complications. This complication rate for revising a reverse is higher than reported rates of complication when revising hemiarthroplasty or anatomic shoulder arthroplasty to a reverse prosthesis.^23^^,^^41^^,^^45^^,^^50^ Authors postulate that in many cases, the indication for the initial reverse surgery is a failed prior arthroplasty thus the increased risk of complication in subsequent interventions.^5^^,^^45^ Notwithstanding the 25% rate of complication, patients reported satisfaction of 89% which demonstrates the improvements in function that are provided by RSA. A similar observation and reasoning were described in 2 of the included studies.^3^^,^^5^ Despite additional procedures which may follow revision RSA, the relief of pain and functional improvement afforded by RSA provides benefit and value to patients.

The current results are consistent with prior reports which detail higher rates of complication for revision shoulder arthroplasty compared with primary shoulder arthroplasty. Wall et al^51^ prospectively analyzed primary and revision reverse with complications rates being 13% and 37%, respectively. Patients undergoing revision did have a gain in function which was comparable to the gain in function in primary patients. Saltzman et al^40^ did a retrospective comparison of outcomes in primary reverse and revision reverse surgery. Revision patients required transfusions at a much higher rate than in primary cases which speaks to the complexities and time requirement of revision surgery. The authors reported a 15% major complication rate among patients undergoing the revision procedure and 9% rate of major complication for those undergoing primary arthroplasty, with no significant difference between these groups. Groh and Groh^24^ reported significant differences in complication rates when comparing primary (4%) and revision (19%).

Given the dearth of reporting, we were unable to perform pooled analysis for the indications which may predispose for certain complications. Of the 4 studies (44%) which detailed major complications, only 2 studies had specific complications which occurred in more than 1 patient. Wagner et al^49^ reported 5 dislocations, representing 45% of his overall complication rate. With 67% of the 27 cases (N = 18) in this series being indicated for revision surgery due to dislocation, we can postulate that the mechanism of instability which resulted in dislocation, may still be present following implantation of a new reverse prosthesis. Similarly, Black et al reported complications of glenoid loosening (N = 3) and instability (N = 3) as 75% of their overall complications.^3^ Instability (N = 6) and glenoid failure (N = 7) represented 82% of the indications in their 16 revision cases. These findings reinforce the lack of certainty when revising RSA to another RSA which needs to be adequately discussed with patients preoperatively. Across the 5 studies which detailed indications for the primary reverse procedure, 4 studies performed RSA for a failed prior hemiarthroplasty or anatomic shoulder arthroplasty. Two of these 4 studies reported more than 30% of failed primary reverses were implanted for failure of a hemiarthroplasty or anatomic shoulder arthroplasty.^3^^,^^5^ In both of these studies, additional surgical revision was burdensome which is in agreement with findings by Stephens et al^44^ of increased failure rates of RSA after a prior failed arthroplasty.

Across the studies which reported the indication for revision surgery, infection represented 43% of revisions.^2^^,^^3^^,^^5^^,^^36^^,^^49^ In revision cases for infection, Beekman et al^2^ and Cuff et al^15^ advocate for a very thorough debridement of infected tissue to reduce the chances of recurrent infection. This extensive debridement can compromise rotator cuff function which would prove problematic for a nonconstrained prosthesis. Given RSA’s reliance on the deltoid for function, the authors were careful to preserve the deltoid while adopting a more aggressive resection of other tissues. With this thorough approach, Beekman et al^2^ reported 10 of 11 patients being infection-free at 24 months.

Instability is one of the most common indications for revision of a primary reverse prosthesis.^5^ In addition, revision shoulder surgery has the intrinsic risk of instability due to altered tissue tension from prior surgical disturbance. RSA offers the unique ability to increase stability via increased component sizing which can mitigate further complications due to instability.^4^^,^^5^^,^^7^^,^^12^^,^^22^ Authors have used larger glenosphere sizing during revision RSA given the characteristic tissue laxity in these procedures. The larger sphere can further stabilize the construct and increase deltoid tension for functional gain.^4^^,^^26^^,^^44^ Furthermore, stability can also be gained by biologic lateralization and by humeral component design.^38^ Biomechanical analysis and simulated modeling have suggested that a lateralized center of rotation increases compressive forces which contribute to improvement in stability.^14^^,^^31^

Cheung et al^12^ reviewed RSA patients for instability in the early postoperative period with results suggesting that subscapularis repair improves stability and lowers rates of dislocation. Several authors admit the potential for nonviable subscapularis tissue in revision cases but if tissue quality permits, they recommend a repair be attempted.^3^^,^^12^^,^^20^

When revising glenoid components, surgeons are faced with the common complication of bony defects. Prior research has demonstrated an association between glenoid defects and poor outcomes in revision shoulder arthroplasty.^18^ Addressing these defects with bone graft may mitigate these complications and yield promising outcomes.^1^^,^^27^^,^^28^^,^^32^^,^^34^^,^^35^^,^^47^^,^^50^ Reports on glenoid bone grafting when revising prior arthroplasty to RSA have shown graft incorporation greater than 90%^28^^,^^50^ though others have attained more inferior results.^27^^,^^35^ Elements of the reversed design which are advantageous for glenoid bone graft incorporation include its multiple points of fixation for adequately securing the graft as well as options to lengthen the center post and peripheral screws.^4^^,^^28^^,^^32^^,^^34^ Compression created by the construct has been correlated with improved graft incorporation.^6^^,^^27^^,^^33^ Wagner et al^47^ suggested glenoid bone graft incorporation may be enhanced by medialized center of rotation reverse constructs given the increased shear across the graft-implant interface with more lateralized designs. Biomechanical analysis of lateralized RSA constructs confirmed an increased shear force, but this increase in shear was less than the increase in compression across the joint which may prove advantageous for graft incorporation.^14^^,^^31^ Wagner et al^47^ concluded glenoid bone grafting in revision RSA was associated with greater rates of glenoid loosening and subsequent failure when compared with cases of revision RSA without glenoid bone graft. Their overall revision rate due to glenoid loosening was 10%. The authors postulated that glenoid bone graft was required in those shoulder with considerably greater glenoid pathologies which predisposes them to complications.

Our findings are limited by the clarity of reporting by others as well as the design of the studies which were analyzed. Some of the identified studies were performed at the same institution which may represent intersection of pooled data. Patient demographics, surgical technique, and gathering of outcome measures are some of the variables that may bias conclusions from a single institution. Variance in outcome measure reporting is another potential limitation. American Shoulder and Elbow Surgeons Shoulder scores and Constant scores were reported by only 5 of 9 studies. Elevation and external rotation were also reported by only 5 studies with abduction being reported in 3 studies. As previously discussed, complications were detailed in 5 of the 9 studies. We hope future reports offer consistent outcome measure reporting and thus, a more cohesive and robust conclusion can be applied. Finally, further evaluation is needed at longer-term follow-ups to fully attest the viability of reverse surgery in this subset of patients.

## Conclusion

With the available short-term data, we can conclude that revising a failed reverse to another reverse is a reasonable treatment option which can yield favorable function, though rates of complication are concerning. More consistent reporting will help future reviews provide more generalizable conclusions. Characteristics of the reverse prosthesis which provide value when revising a reverse prosthesis include its strength of glenoid fixation for bone grafting, the ability to increase construct stability and its reliance on the deltoid for function. As our learning about revision of RSA improves, so will our ability to preemptively address potential issues which may lead to decreased complications in these cases.

## Disclaimers

Funding: No funding was disclosed by the author(s).

Conflicts of interest: Dr. Wagner receives consulting fees from Stryker, Wright Medical, Biomet, Acumed, and Osteoremedies, and research support from Arthrex, Konica Minolta, Arthrex, and DJO. None are relevant to this manuscript.

The other authors, their immediate family, and any research foundation with which they are affiliated have not received any financial payments or other benefits from any commercial entity related to the subject of this article.
